# Advanced Quantification of Receptor–Ligand Interaction Lifetimes via Single-Molecule FRET Microscopy

**DOI:** 10.3390/biom14081001

**Published:** 2024-08-13

**Authors:** Lukas Schrangl, Vanessa Mühlgrabner, René Platzer, Florian Kellner, Josephine Wieland, Reinhard Obst, José L. Toca-Herrera, Johannes B. Huppa, Gerhard J. Schütz, Janett Göhring

**Affiliations:** 1 Institute of Biophysics, Department of Bionanosciences, University of Natural Resources and Life Sciences, Muthgasse 11, 1190 Vienna, Austria; lukas.schrangl@boku.ac.at (L.S.);; 2 Institute of Applied Physics, TU Wien, Wiedner Hauptstr. 8-10, 1040 Vienna, Austria; 3 Institute for Hygiene and Applied Immunology, Center for Pathophysiology, Infectiology and Immunology, Medical University of Vienna, Lazarettgasse 19, 1090 Vienna, Austria; 4 Institute for Immunology, Biomedical Center, Medical Faculty, Ludwig-Maximilians-Universität München, 82152 Planegg-Martinsried, Germany

**Keywords:** single-molecule FRET, single-molecule microscopy, receptor-ligand interaction, T cell receptor, bond lifetime quantification, T cell activation, antigen sensitivity, simulation, survival analysis

## Abstract

Receptor–ligand interactions at cell interfaces initiate signaling cascades essential for cellular communication and effector functions. Specifically, T cell receptor (TCR) interactions with pathogen-derived peptides presented by the major histocompatibility complex (pMHC) molecules on antigen-presenting cells are crucial for T cell activation. The binding duration, or dwell time, of TCR–pMHC interactions correlates with downstream signaling efficacy, with strong agonists exhibiting longer lifetimes compared to weak agonists. Traditional surface plasmon resonance (SPR) methods quantify 3D affinity but lack cellular context and fail to account for factors like membrane fluctuations. In the recent years, single-molecule Förster resonance energy transfer (smFRET) has been applied to measure 2D binding kinetics of TCR–pMHC interactions in a cellular context. Here, we introduce a rigorous mathematical model based on survival analysis to determine exponentially distributed receptor–ligand interaction lifetimes, verified through simulated data. Additionally, we developed a comprehensive analysis pipeline to extract interaction lifetimes from raw microscopy images, demonstrating the model’s accuracy and robustness across multiple TCR–pMHC pairs. Our new software suite automates data processing to enhance throughput and reduce bias. This methodology provides a refined tool for investigating T cell activation mechanisms, offering insights into immune response modulation.

## 1. Introduction

A cell’s means of communicating with the outside world depends on the screening and specific binding to molecular recognition patterns in order to react to or impact the cellular environment. Hence, the molecular binding dynamics of receptor–ligand pairs at the interface of cells are at the very beginning of many signaling cascades leading to essential effector functions.

Receptor–ligand interactions can be discriminated into the interaction of soluble ligands with their respective membrane-associated receptors (3D environment), or the interaction of ligands attached to a solid phase, be it another cellular membrane or extracellular components (2D environment). The immune system has a variety of highly motile cells in its arsenal in order to scan for sources of antigen. One of these prominent receptor–ligand interactions are essential for the effector functions of T cells, a key player of the adaptive immune system. Pathogen-derived peptides are presented by the major histocompatibility complex (pMHC) proteins at the surfaces of antigen-presenting cells, while T cells scan for these complexes via their T cell receptors (TCRs).

The binding duration (or dwell time) of the pMHC complex to the TCR is a direct reflection of its affinity and other physicochemical parameters. Affinity [1] as well as on- and off-rates [2] are in line with quantitative parameters of the downstream signaling cascades leading to T cell activation. Strong agonistic interactions often exhibit several seconds to tens of seconds length, whereas weak agonists can feature lifetimes of less than 100 ms, as shown for CD4+ [1] and CD8+ [3] T cells. The rates of unbinding are therefore relevant read-out parameters at the state of signal initiation for a multitude of immunological assays. This is especially important since the mere measurement of 3D affinity is often not enough to predict the stimulatory potency of antigenic peptides. Additional aspects such as binding geometry [4], mechanical forces [5], and membrane geometry and composition as well as binding cooperativity [6] need to be considered.

The standard approach for quantification of affinity and binding kinetics utilizes surface plasmon resonance (SPR) to study the interaction of purified recombinant TCRs and pMHCs of interest [4,6]. This method sensitively quantitates the 3D affinity of the interaction partners, but does not provide any cellular context and hence does not encompass cellular interactions such as adhesion factors, co-stimulation, or coreceptor engagement. Especially, the impact of external forces, membrane fluctuation and protein flexibility cannot be estimated by SPR [6]. An alternative approach for receptor–ligand interaction lifetime quantification in 2D uses fluorescence microscopy and is based on single-molecule Förster resonance energy transfer (smFRET) [7]. In this method, a functionalized glass-supported lipid bilayer (SLB) is used as mimicry of the surface of an antigen-presenting cell. Besides adhesion and co-stimulation factors, it contains MHCs loaded with fluorescently labeled peptides that act as FRET acceptors; the corresponding TCRs of SLB-interacting T cells are fluorescently labeled with a specific antibody fragment providing the FRET donor molecule. Whenever the two binding partners engage, the donor molecule transfers its energy to the acceptor molecule and a FRET signal can be observed. From multiple single-molecule FRET signals the interaction lifetime of the receptor–ligand pair can be quantified. The 2D binding kinetics measured in such a way are in line with the downstream signaling outcome [7,8].

This study introduces a rigorous mathematical model for determination of exponentially distributed receptor–ligand interaction lifetimes based on the statistical framework of survival analysis. The model is verified and characterized using simulated data. Additionally, a comprehensive analysis pipeline to infer interaction lifetimes from raw microscopy image data is presented and applied to experimental data from several TCR–pMHC pairs.

The new algorithm yields accurate and robust results and allows for determination of TCR–pMHC interaction lifetimes across several orders of magnitude. Furthermore, we created a new analysis software suite [9] which strives to rely on automated processing as much as possible to optimize throughput and to minimize subjective criteria which could lead to biased data presentation.

## 2. Materials and Methods

### 2.1. Animal Model

5c.c7 αβ TCR-transgenic mice (Tg(Tcra5CC7,Tcrb5CC7)IWep, PMID: 1328464) bred onto the B10.A background were a kind gift from Michael Dustin (University of Oxford, UK). The mice were housed in groups of 2–5 per cage in the pathogen-free facility at the Medical University of Vienna, Austria. Spleens and lymph nodes were harvested from 12–16 weeks old gender-mixed mice.

Spleens of AND-TCR transgenic B10.BR animals (Tg(TcrAND)53Hed, PMID: 2571940) were removed and sent in Dulbeccos’s Modified Eagle’s Medium (DMEM)/1% Bovine serum albumin (BSA) to the Medical University of Vienna on ice. The mice were genotyped by polymerase chain reaction (PCR) or by cytometry and housed in groups of 2–5 animals per cage in the specific pathogen-free Core Facility Animal Models at the Biomedical Center of LMU Munich, Germany.

### 2.2. Tissue Culture

Splenocytes or lymphocytes of 5c.c7 and AND αβ TCR-transgenic mice were isolated and pulsed with 2 µM C18 reverse-phase high-performance liquid chromatography (HPLC)-purified moth cytochrome C (MCC) (88-103) peptide (sequence: ANERADLIAYLKQATK; Intavis, Tübingen, Germany) and 50 U mL^−1^ IL-2 (eBioscience, San Diego, CA, USA) for 7 days to arrive at a transgenic T cell culture [10]. T cells were maintained at 37 °C in an atmosphere of 5% CO_2_ in RPMI 1640 media (Life technologies, Carlsbad, CA, USA) supplemented with 100 µg mL^−1^ penicillin (Life technologies, Carlsbad, CA, USA), 100 µg mL^−1^ streptomycin (Life technologies, Carlsbad, CA, USA), 2 mM L-glutamine (Life technologies, Carlsbad, CA, USA), 10% fetal calf serum (FCS; Biowest, Nuaillé, France), 0.1 mM non-essential amino acids (Lonza, Basel, Switzerland), 1 mM sodium pyruvate (Life technologies, Carlsbad, CA, USA) and 50 µM β-mercaptoethanol (Life technologies, Carlsbad, CA, USA). After expansion, debris and dead cells were removed by Histopaque-1119 (Merck KGaA, Darmstadt, Germany) density gradient centrifugation. Antigen-experienced T cells were used for experiments from day eight to ten.

### 2.3. Formation of Functionalized Lipid Bilayers

Lipids dissolved in chloroform were mixed (98 mol-% POPC (1-palmitoyl-2-oleoyl-glycero-3-phosphocholine) plus 2 mol-% DGS-NTA(Ni) (1,2-dioleoyl-sn-glycero-3-[(N-(5-amino-1-carboxypentyl)iminodiacetic acid)succinyl] (nickel salt)); Avanti Polar Lipids, Inc., Alabaster, AL, USA) and subsequently dried under a nitrogen stream for 20 min in a glass test tube (Schott FIOLAX 12 × 75 mm, Carl Roth, Karlsruhe, Germany). After resuspension in 1 mL Dulbecco’s Phosphate Buffered Saline (DPBS; Merck KGaA, Darmstadt, Germany), they were sonicated for 10 min in an ultrasound water bath (USC500TH, VWR, Lutterworth, UK) at room temperature. The resulting small unilamellar vesicle solution was diluted to 125 µM using DPBS.

The original cover slip of an eight-well chamber (Nunc Lab-Tek, Thermo Fisher Scientific, Waltham, MA, USA) was replaced by attaching a plasma-treated (10 min; PDC-002 Plasma Cleaner, Harrick Plasma Inc, Ithaca, NY, USA) microscopy cover slip (MENZEL-Gläser Deckgläser 24 × 60 mm #1.5) using duplicating silicone (Twinsil soft 18, picodent, Wipperfürth, Germany). 150 µL of vesicle solution was filled into each well and left to incubate for 20 min at room temperature. Subsequent washing with DPBS removed excess vesicles.

Into each well, 30 ng of histidine (his)-tagged murine intercellular adhesion molecule (ICAM) 1, 50 ng of his-tagged murine B7-1, and 2 ng of his-tagged IE^k^-MCC-Alexa Fluor 647 (for experiments with murine T cells) were added and left for incubation for 75 min. Unbound excess proteins were washed away using DPBS.

### 2.4. Protein Expression and Refolding

The TCR β-reactive H57 single-chain fragment (scF_V_) (J0, GenBank: MH045460.1) and the fluorescently labelled H57 scF_V_ (J1, GenBank: MH045461.1) were produced as described [11]. In short, scF_V_ constructs were expressed in Escherichia coli and inclusion bodies were extracted. H57 scF_V_s were refolded in vitro, concentrated, and purified by gel filtration. The monomeric H57 scF_V_ (J1) was conjugated with Alexa Fluor 555 C2 Maleimide (Thermo Fisher Scientific, Waltham, MA, USA). Protein-to-dye ratios of site-specifically decorated H57 scF_V_-Alexa Fluor 555 were 1.0.

The murine MHC class II molecule IE^k^ α subunits (with a 12× histidine-tag) and the β subunits were expressed in *E. coli* as inclusion bodies and refolded in vitro with a fluorescently labelled MCC peptide as described [11]. In short, his-tagged IE^k^/MCC was refolded and purified via nickel–nitrilotriacetic acid (Ni-NTA)-based affinity chromatography followed by gel filtration. MCC peptides were site-specifically labelled via Alexa Fluor 647 C2 Maleimide (Thermo Fisher Scientific, Waltham, MA, USA) and purified as described [11].

Murine recombinant ICAM-1-10xHis and B7-1-10xHis were purchased from Sino Biological (Beijing, China).

### 2.5. Single-Molecule Fluorescence Microscopy

By means of an objective with high numerical aperture (α Plan-FLUAR 100×/1.45 oil, Carl Zeiss GmbH, Oberkochen, Germany), objective-type total internal reflection (TIR) illumination of fluorophores was realized. Donor fluorophores (Alexa Fluor 555, Life technologies, Carlsbad, CA, USA) were excited with a 532 nm laser (LCX-532L with L1C-AOM, Oxxius, Lannion, France), acceptor fluorophores (Alexa Fluor 647, Life technologies, Carlsbad, CA, USA) with a 640 nm laser (OBIS 640, Coherent Inc., Saxonburg, PA, USA). A quad-band dichroic mirror (Di01-R405/488/532/635-25 × 36, Semrock part of IDEX Health & Science, LLC, Rochester, NY, USA) separated the emission beam from the excitation light. With the help of a beam splitter device (Optosplit II, Cairn Research, Faversham, UK) employing a dichroic mirror (FF640-FDi01-25 × 36, Semrock part of IDEX Health & Science, LLC, Rochester, NY, USA) and bandpass filters (ET570/60m and ET675/50m, Chroma Technology Corp, Bellows Falls, VT, USA), donor and acceptor fluorophore emission images were projected side by side onto the chip of an electron multiplying charge-coupled device (EM-CCD) camera (Andor iXon Ultra 897, Andor Technology Ltd., Belfast, UK). The microscope and peripherals were controlled by using the SDT-control software (version 2.18, developed in-house).

Stroboscopic illumination of fluorophores was synchronized with camera read-out. For the main illumination sequence, the following operations were repeated a pre-defined number of times: (a) read out of the camera chip to remove charges from stray light, (b) excitation of donor fluorophores and simultaneous recording of donor and acceptor emission, (c) pause for a pre-defined amount of time to achieve the desired recording rate.

The number of repeats was typically chosen to allow monitoring a single cell for several minutes. Before and after this sequence, a single image upon acceptor excitation was recorded to assess the integrity of the lipid bilayer.

TCRs were fluorescently labeled as follows: $10^{6}$ cells in medium were centrifuged for 3 min at 350 g. Consecutively, the supernatant was removed, leaving the cells in roughly 60 µL of medium. 45 ng of unlabeled and 15 ng of fluorescently labeled H57-scF_V_ were added in 5 µL of DPBS and left for incubation on ice for 20 min. Excess scF_V_ was washed away by adding 5 mL of Hank’s Buffered Salt Solution (HBSS; Merck KGaA, Darmstadt, Germany) + 2% fetal calf serum (FCS; Merck KGaA, Darmstadt, Germany) at 4 °C, centrifuging for 3 min at 350 g and 4 °C and removing the supernatant. Cells were subsequently kept on ice.

Immediately before microscopy measurements, the buffer in the wells containing SLBs was exchanged for HBSS + 2% FCS (pre-warmed to the temperature of interest). $10^{5}$ T cells were added and left to attach for 2–3 min.

### 2.6. Calcium Flux Measurements

T cell quality was monitored via calcium flux experiments in parallel to the single-molecule FRET experiments. Intracellular calcium levels were measured with the ratio-metric dye Fura-2-AM (Thermo Fisher Scientific, Waltham, MA, USA) as published [12]. 1–2 $\times \;10^{6}$ T cells were incubated with 2 µM Fura-2-AM (Life technologies, Carlsbad, CA, USA) in T cell growth medium for 15–20 min at room temperature and subsequently washed with warm (room temperature) imaging buffer (1x HBSS (Life technologies, Carlsbad, CA, USA) supplemented with 2% FCS (Biowest, Nuaillé, France), 2 mM CaCl_2_ and 2 mM MgCl_2_ (MerckKGaA, Darmstadt, Germany)). Immediately afterwards, T cells were seeded onto functionalized SLBs featuring either unlabeled B7-1 and ICAM-1 (100 µm^−2^) as negative control, or additionally unlabeled IE^k^/MCC (100 µm^−2^) as positive control. Calcium response was recorded at room temperature. Fura-2 was excited with a monochromatic light source (Polychrome V, TILL Photonics, Gräfelfing, Germany) coupled to a Zeiss Axiovert 200 M equipped with a 10× objective (UPlanFL N 10×, NA = 0.3, Olympus, Tokyo, Japan), a 1.6× tube lens, a long-pass filter (T400lp, Chroma Technology Corp, Bellows Falls, VT, USA), an emission filter ET510/80 (Chroma Technology Corp, Bellows Falls, VT, USA) and an EM-CCD camera (Andor iXon 897, Andor Technology Ltd., Belfast, UK). Imaging was performed with excitation at 340 and 380 nm with illumination times of 50 and 10 ms, respectively. The total recording time was 10 min with one image per second. Calcium image analysis was carried out with a custom software written in the MATLAB (Mathworks, Inc., Natick, MA, USA) language [13].

### 2.7. Maximum Likelihood Estimation (MLE) of Apparent Lifetimes

For the inference of apparent lifetimes from single-molecule track lengths by means of survival analysis, the lifelines Python package [14] was used. Maximizing the likelihood derived in Section 3.1 is implemented in the lifelines.ExponentialFitter class via the fit_interval_censoring method, which yields the maximum likelihood estimate and its standard error.

To perform “conventional” MLE (i.e., no proper handling of traces continuing beyond the observation window), we calculated the mean track length shifted by $t_{\min}$. The result was additionally shifted by half a recording interval to account for the fact that the signal vanishes sometime between two recordings. This results in $\tau_{\mathrm{app}}=\mathrm{mean}\left\{t_{i}-t_{\min}\right\}+0.5\Delta t$ using the nomenclature from Section 3.1.

### 2.8. Fitting of the Characteristic Lifetime

Having inferred apparent lifetimes at different recording intervals, Equation (1) was fit by means of a non-linear least squares method using the optimize.curve_fit function from the SciPy Python package [15]. Apparent lifetimes’ standard errors were used as weights for the fit. To estimate the uncertainty of the fit results, the square roots of the resulting covariance matrix’s diagonal elements was computed. The lower and upper bound of the uncertainty band in apparent lifetime vs. recording interval plots were computed via Equation (1) by substituting fit results minus and plus standard errors, respectively.

### 2.9. Simulation of Single-Molecule FRET Time Traces

Generation of on- and off-state changes of the FRET signals proceeded in the same manner as simulation of state transition trajectories described previously [16]. In short, durations of on-state and off-state were randomly drawn from exponential distributions with decay time parameters $\tau_{\mathrm{app}}$ and $\tau_{\mathrm{off}}$. These durations were concatenated to form a trajectory. $\tau_{\mathrm{app}}$ was set according to Equation (1) with $c_{b}=30$, which is in the typical range of values determined from experiments, $\tau_{\mathrm{lt}}=10\;s$, and Δt as indicated in Section 3.2. $\tau_{\mathrm{off}}$ was fixed as $10^{3}\;s$.

Next, the trajectories were sampled at fixed intervals Δt for a duration $t_{\mathrm{obs}}$ (observation window) to simulate stroboscopic illumination, starting after a predefined time (2000s) to allow for transients to subside. The number of consecutive samples in which a trajectory was in the on-state, corresponding to the number of frames a FRET signal was visible in an experiment (termed $n_{i}$ in Section 3.1), was used to test our method (Section 3.2). The number of sampling points $n_{\mathrm{samp}}$ (see Supplementary Tables S1 and S2 for the respective values), and thus the observation window $t_{\mathrm{obs}}=n_{\mathrm{samp}}\Delta t$, was adjusted for each Δt to yield more trajectories present at the beginning or at the end of the measurement window, and fewer that fully lie within the window than in a typical experiment (see Supplementary Figures S1–S3) to further challenge our algorithm.

### 2.10. Microscopy Data Analysis

Extraction of single-molecule FRET time traces from raw microscopy images relied heavily on methods provided by the sdt Python package [17]. The sdt.roi.ROI class allowed for defining the emission channels within raw images. The single-molecule localization algorithm [18] implemented in the sdt.loc.cg module was used to detect fluorescent beads for image registration as well as the single-molecule FRET signals. Signal intensities were measured by summing pixel intensity values in a region around respective positions via the sdt.brightness.from_raw_image function. Image registration was done using the sdt.multicolor.Registrator class. The trackpy Python package [19] was utilized to perform single-molecule tracking. Each track was extended by 5 frames before the first and after the last frame by measuring the intensity at the first and last position of the track, respectively. This permitted employing a changepoint detection algorithm (PELT [20] as implemented in the sdt.changepoint module) for stepwise bleaching analysis of smFRET signals.

All analysis steps are integrated into our software GUI application, which leverages functionality from the sdt.gui sub-package. Plots were created using the matplotlib Python package [21].

## 3. Results

**Figure 1:**
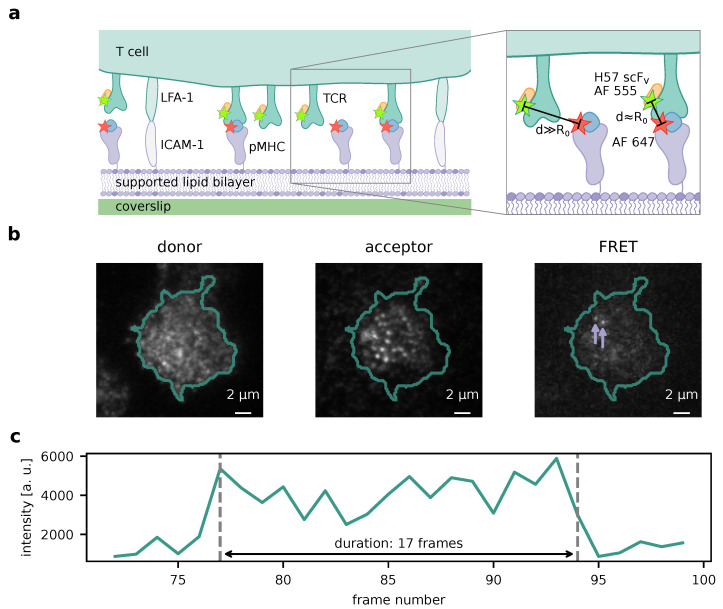
Experimental method. (**a**) Measurement of TCR–pMHC interaction times. T cell receptors are labeled using an H57 scF_V_ carrying the FRET donor fluorophore (Alexa Fluor (AF) 555). A functionalized SLB carrying adhesion proteins (ICAM-1), co-stimulatory molecules (B7-1, not shown), and pMHC acts as an antigen presenting cell surrogate. The pMHC presents a stimulatory peptide labeled with the FRET acceptor fluorophore (Alexa Fluor (AF) 647). Only when a ligand is bound to a receptor, fluorophores are close enough (separated by about their Förster radius $R_{0}$) to enable FRET. (**b**) Resulting microscopy image data. Left: Emission of donor fluorophores (TCR labels) upon donor excitation (beginning of the recording). Cell contours were determined via adaptive thresholding. Center: Acceptor fluorophores labeling SLB-bound pMHC upon acceptor excitation (beginning of the recording). Right: FRET signals (acceptor emission upon donor excitation), indicating TCR–pMHC bond, are pointed out by the arrows (77th frame of the recording). (**c**) Exemplary single-molecule FRET time trace. The time trace of the rightmost signal from (**b**) appears and vanishes in single, discrete steps and exhibits a plateau, suggesting single-molecular origin.

This study presents a new approach for extraction of average lifetime estimates from single molecule FRET microscopy data. To exploit FRET for the determination of receptor–ligand interaction lifetimes, receptors need to be fluorescently labeled with one constituent of a FRET pair, while ligands need to carry the other constituent. Fluorophores, as well as their attachment sites and -strategies, have to be chosen such that their spatial separation is less than or roughly equal to the Förster radius $R_{0}$ when receptor and ligand are bound. Figure 1a illustrates this for TCR and pMHC as interaction partners, for which bond lifetimes have been successfully measured [7]. If binding events are rare enough, they appear as single-molecule FRET events (Figure 1b), which can be traced over time and allow for measurement of their durations (Figure 1c).

Single-molecule tracks may, however, not only end because of receptor–ligand unbinding, but also because of photobleaching of one of the fluorophores forming the FRET pair. Since labeling density is high in both the donor and the acceptor channel (Figure 1b), it is not possible to separate the causes directly e.g., by means of alternating laser excitation [22]. Instead, we rely on the observation that the interaction lifetime is independent of the fluorophore illumination, while the photobleaching rate is not. Hence, the true characteristic lifetime can be inferred from datasets recorded with different intervals between consecutive frames [7].

In the following, we present

- a mathematical model for accurate determination of binding lifetimes from single-molecule tracking data (Section 3.1),
- characterization of the model by evaluating simulated data and comparing the results to the known ground truth (Section 3.2),
- an efficient data analysis pipeline (Section 3.3), which we implemented in an easy-to-use, free and open source software application, and
- application of said analysis pipeline to experimental data from TCR–pMHC pairs with lifetimes spanning several orders of magnitude (Section 3.4).

### 3.1. Mathematical Framework

We model the bond lifetimes $T_{\mathrm{lt}}$ as exponentially distributed random variables, $T_{\mathrm{lt}}∼\mathrm{Exp}\left(\lambda_{\mathrm{lt}}\right)$ [7,23]. Fluorophore survival times with respect to photobleaching $T_{b}$ are also assumed to be exponentially distributed, $T_{b}∼\mathrm{Exp}\left(\lambda_{b}\right)$. Since these are competing processes, the apparent lifetimes $T_{\mathrm{app}}$ measured in the experiment are exponentially distributed as well, $T_{\mathrm{app}}∼\mathrm{Exp}\left(\lambda_{\mathrm{lt}}+\lambda_{b}\right)$.

When using stroboscopic illumination, i.e., fluorophores are only excited during the image acquisitions, but not in between, the photobleaching rate $\lambda_{b}$ is proportional to the acquisition rate and therefore inversely proportional to the interval Δt between images. Mathematically, $\lambda_{b}=\frac{1}{c_{b}\Delta t}$ for some constant $c_{b}$. The characteristic apparent lifetime $\tau_{\mathrm{app}}:=\frac{1}{\lambda_{\mathrm{lt}}+\lambda_{b}}$ is thus a function of Δt,

(1) $$\tau_{\mathrm{app}}\left(\Delta t\right)=\frac{1}{\frac{1}{\tau_{\mathrm{lt}}}+\frac{1}{c_{b}\Delta t}},$$

where $\tau_{\mathrm{lt}}:=\frac{1}{\lambda_{\mathrm{lt}}}$ is the characteristic bond lifetime. With apparent lifetimes $\tau_{\mathrm{app}}$ inferred from tracking data recorded at various Δt, $\tau_{\mathrm{lt}}$ (and also $c_{b}$) can therefore be determined by fitting Equation (1).

For fixed Δt, the single-molecule FRET tracking experiment yields a set of frame counts $n_{i}$, which is defined as the number of frames the *i*-th bound ligand’s signal is counted within the recording window. In order to accurately determine the characteristic apparent lifetime $\tau_{\mathrm{app}}\left(\Delta t\right)$, the following aspects need to be considered:

1. The exact moment of unbinding is unknown. If a signal is detectable until the *j*-th frame (j<lastframe), whereafter it disappears, the unbinding / bleaching time point lies between the *j*-th and the (j+1)-th frame. A signal can also be still present at the end of a recording.
2. The exact time of binding is unknown. If a signal first appears in the *j*-th frame (j>1), the time of binding lies between the (j−1)-th and the *j*-th frame. A signal can also be already present at the start of a recording.
3. To distinguish actual single-molecule FRET tracks from short-lived noise e.g., due to cellular background fluctuations, one may wish to introduce a minimum length $n_{\min}$ for tracks to analyze.

We employ survival analysis to take these issues into account. For a comprehensive introduction to survival analysis, refer e.g., to Klein and Moeschberger [24]. In order to infer the $\tau_{\mathrm{app}}$, we perform maximum likelihood estimation. To this end, the log-likelihood

(2) $$\log\ell \left(\tau \right)=\sum_{i}\log\ell_{i}\left(\tau \right)$$

is maximized with respect to τ, where $\ell_{i}\left(\tau \right)$ is the likelihood contribution of the *i*-th single-molecule track’s frame count $n_{i}$. τ denotes the characteristic decay time (i.e., the inverse of the rate parameter) of the exponential distribution.

Considering only the first aspect in the list above, a track detected for a time $t_{i}:=\left(n_{i}-1\right)\Delta t$ yields

(3) $$\ell_{i}^{\left(1\right)}\left(\tau \right)=P\left(t_{i}\leq T_{\mathrm{app}}\leq t_{i}+\Delta t;\tau \right)$$

if the signal ceased before the end of the observation window and

(4) $$\ell_{i}^{\left(1\right)}\left(\tau \right)=P\left(t_{i}\leq T_{\mathrm{app}};\tau \right)$$

otherwise.

**Figure 2:**
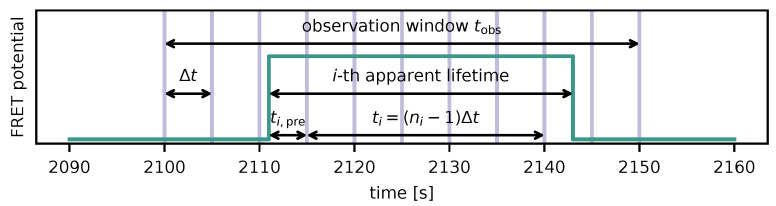
Illustration of variables defined for survival analysis. The green line indicates a potential smFRET trace. Within the observation window, microscopy images are recorded repeatedly at intervals Δt, depicted by purple vertical lines. A binding event takes place some time $t_{i,\mathrm{pre}}$ before being recorded in a microscopy frame. After its apparent lifetime, which we interpret as a realization of the (exponentially distributed) random variable $T_{\mathrm{app}}$, FRET is terminated as a result of unbinding or photobleaching. The measured duration $t_{i}$ is derived from the number of frames $n_{i}$ in which the smFRET signal was detectable.

Receptor and ligand generally start to interact an unknown time $t_{i,\mathrm{pre}}$ before the first exposure (aspect #2; see also Figure 2), which needs to be added to $t_{i}$ (i.e., $t_{i}\mapsto t_{i}+t_{i,\mathrm{pre}}$) in the equations above. The fact that the receptor–ligand pair would escape detection if its binding duration was shorter than $t_{i,\mathrm{pre}}$ needs to be accounted for by probabilities being conditional on $T_{\mathrm{app}}\geq t_{i,\mathrm{pre}}$. The *i*-th likelihood contribution accommodating both aspects #1 and #2 is thus given by

(5) $$\ell_{i}^{\left(1,2\right)}\left(\tau \right)=P\left(t_{i}+t_{i,\mathrm{pre}}\leq T_{\mathrm{app}}\leq t_{i}+t_{i,\mathrm{pre}}+\Delta t|T_{\mathrm{app}}\geq t_{i,\mathrm{pre}};\tau \right)$$

for tracks disappearing during the measurement and

(6) $$\ell_{i}^{\left(1,2\right)}\left(\tau \right)=P\left(t_{i}+t_{i,\mathrm{pre}}\leq T_{\mathrm{app}}|T_{\mathrm{app}}\geq t_{i,\mathrm{pre}};\tau \right)$$

for tracks still present at the end.

Since the exponential distribution is memory-less, i.e., P(T>x+y∣T>x)=P(T>y), these expressions reduce to

(7) $$\ell_{i}^{\left(1,2\right)}\left(\tau \right)=P\left(t_{i}\leq T_{\mathrm{app}}<t_{i}+\Delta t;\tau \right)\;\mathrm{and}\;\ell_{i}^{\left(1,2\right)}\left(\tau \right)=P\left(t_{i}\leq T_{\mathrm{app}};\tau \right),$$

respectively.

The minimum frame count $n_{\min}$ (aspect #3) is taken into account by modifying the expressions above to be conditional on $T_{\mathrm{app}}\geq t_{\min}:=\left(n_{\min}-1\right)\Delta t$. The full expressions for the *i*-th likelihood contribution are therefore as follows:

(8) $$\ell_{i}\left(\tau \right)=P\left(t_{i}\leq T_{\mathrm{app}}<t_{i}+\Delta t|T_{\mathrm{app}}\geq t_{\min};\tau \right)=\frac{P(t_{i}\leq T_{\mathrm{app}}<t_{i}+\Delta t;\tau )}{P(T_{\mathrm{app}}\geq t_{\min};\tau )}$$

for tracks terminating before the end of the observation window and

(9) $$\ell_{i}\left(\tau \right)=P\left(t_{i}\leq T_{\mathrm{app}}|T_{\mathrm{app}}\geq t_{\min};\tau \right)=\frac{P(t_{i}\leq T_{\mathrm{app}};\tau )}{P(T_{\mathrm{app}}\geq t_{\min};\tau )}$$

for tracks outliving the observation window.

The exponential distribution’s cumulative distribution function (CDF) $F\left(t_{i};\tau \right)=1-\exp\left(-\frac{t_{i}}{\tau }\right)$ can be used together with the identity $P\left(T_{\mathrm{app}}\leq t_{i};\tau \right)=F\left(t_{i};\tau \right)$ to arrive at the functional dependencies

(10) $$\ell_{i}\left(\tau \right)=\left(\exp\left(-\frac{t_{i}}{\tau }\right)-\exp\left(-\frac{t_{i}+\Delta t}{\tau }\right)\right)\exp\left(\frac{t_{\min}}{\tau }\right)\;(\mathrm{vanishing}\;\mathrm{tracks})\;\mathrm{and}$$

(11) $$\ell_{i}\left(\tau \right)=\exp\left(-\frac{t_{i}}{\tau }\right)\exp\left(\frac{t_{\min}}{\tau }\right)\;(\mathrm{surviving}\;\mathrm{tracks}).$$

Performing experiments employing a recording interval Δt yields a set of values $t_{i}$ which, using the expressions above, define the (log) likelihood function $\log\ell \left(\tau \right)=\sum_{i}\log\ell_{i}\left(\tau \right)$. Numerical methods (see Section 2.7) allow for maximizing this likelihood with respect to τ, i.e., finding the value $\tau_{\mathrm{app}}^{\mathrm{MLE}}$ for which logℓ(τ) is maximal. $\tau_{\mathrm{app}}^{\mathrm{MLE}}$ is the maximum likelihood estimate of the characteristic apparent lifetime $\tau_{\mathrm{app}}$. Repeating this process for multiple values of Δt yields pairs $\left(\Delta t,\tau_{\mathrm{app}}^{\mathrm{MLE}}\left(\Delta t\right)\right)$, which permit inference of the interaction lifetime $\tau_{\mathrm{lt}}$ by fitting Equation (1).

### 3.2. Characterization Using Simulated Data

**Figure 3:**
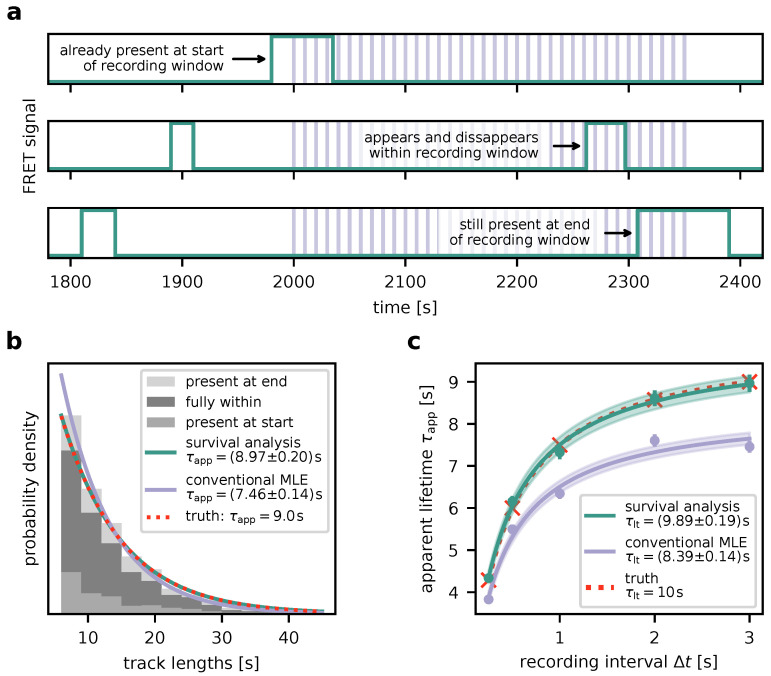
Proof of concept using simulated data. (**a**) Simulation of time traces (green). FRET signals are switched on and off with exponentially distributed lifetimes. Microscopy image acquisitions correspond to sampling the time trace at time points separated by an interval Δt, illustrated by the vertical lines. Possible scenarios taken into account via survival analysis (Section 3.1) are indicated by the annotated arrows. (**b**) Inference of $\tau_{\mathrm{app}}$. The histogram depicts simulated track lengths for fixed Δt=3s for three scenarios: (i) signals are present at the start of the recording window, (ii) at the end of the recording window, (iii) they lie fully within the recording window. The probability density function (PDF) derived using survival analysis (green line) is virtually indistinguishable from the true PDF (red dotted line). Analysis utilizing conventional maximum likelihood estimation (MLE, see also Section 2.7) yields a clear deviation in the PDF (purple line) and a value for $\tau_{\mathrm{app}}$ which is too low. (**c**) Determination of the binding lifetime $\tau_{\mathrm{lt}}$. Datasets as in (**b**) were simulated and analyzed for different Δt. Red crosses mark the simulated values for $\tau_{\mathrm{app}}$, green dots indicate values determined using survival analysis, and purple dots denote values inferred via conventional MLE. Equation (1) was fit to the resulting $\tau_{\mathrm{app}}\left(\Delta t\right)$, of which results are shown as dotted red line, green line, and purple line, respectively. The shaded areas indicate corresponding error margins.

In order to test and characterize our algorithm, we simulated smFRET time traces and analyzed them as described in the previous section. The traces were generated by switching molecules between dark and bright states with exponentially distributed lifetimes characterized by $\tau_{\mathrm{off}}$ and $\tau_{\mathrm{app}}$, respectively. The traces were sampled at a predefined number of discrete time points separated by Δt after an initial time to simulate the image acquisition process. This process is depicted in Figure 3a. For further information as well as the numerical parameter values used, refer to methods Section 2.9.

As a proof of concept, we generated a large dataset (about 2500 FRET traces per Δt, i.e., 25 times the size of a typical experimental dataset) to keep the influence of random fluctuations low. Figure 3b shows a histogram of the track lengths at a single Δt=3s. Employing survival analysis, we were indeed able to infer an accurate estimate $\tau_{\mathrm{app}}=\left(8.97\pm 0.20\right)\;s$ of the true value $\tau_{\mathrm{app}}=9.0\;s$. Notably, attempting to determine the apparent lifetime solely from frame counts—i.e., disregarding the fact that traces may live beyond the observation window (conventional MLE, see Section 2.7 for details)—can lead to a bias towards shorter lifetimes ($\tau_{\mathrm{app}}=\left(7.46\pm 0.14\right)\;s$). From datasets generated at several different Δt (Figure 3c), an accurate estimate for the characteristic interaction lifetime $\tau_{\mathrm{lt}}=10\;s$ could be obtained if apparent lifetimes $\tau_{\mathrm{app}}\left(\Delta t\right)$ were inferred using survival analysis ($\tau_{\mathrm{lt}}=\left(9.89\pm 0.19\right)\;s$). Failure of properly handling traces exceeding the observation window is cause for underestimation ($\tau_{\mathrm{lt}}=\left(8.39\pm 0.14\right)\;s$)).

**Figure 4:**
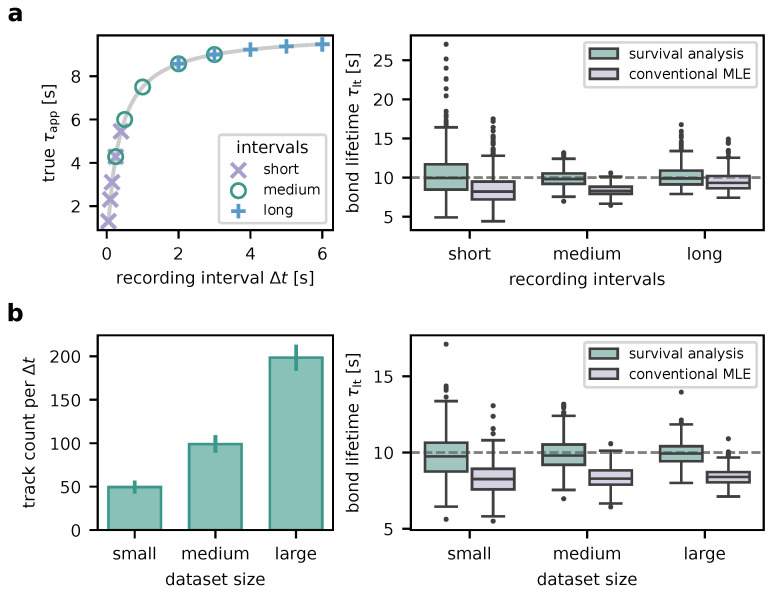
Method robustness characterized using simulated data. (**a**) Choice of recording intervals Δt. Three exemplary sets of recording intervals were chosen. Their location on the $\tau_{\mathrm{app}}$ vs. Δt curve (Equation (1)) is shown in the left panel. The boxplots in the right panel summarize the bond lifetimes $\tau_{\mathrm{lt}}$ as determined from 500 simulated experiments with respective Δt sets. Analysis was performed using both our survival analysis-based method and conventional MLE. The ground truth $\tau_{\mathrm{lt}}=10\;s$ is indicated by the dashed line. (**b**) Dataset size. Using the medium Δt set from (**a**), experiments yielding varying numbers of FRET time traces were simulated (500 experiments per size category). The mean numbers of traces per recording interval are indicated in the left panel (error bars: standard deviations). Bond lifetimes $\tau_{\mathrm{lt}}$ inferred from the datasets are charted in the right panel. As in (**a**), our new method is compared to conventional MLE. The ground truth $\tau_{\mathrm{lt}}=10\;s$ is plotted as a dashed line.

Next, we wanted to explore the impact of the choice of recording intervals Δt with respect to the a priori unknown interaction lifetime $\tau_{\mathrm{lt}}$. To this end, we simulated 500 experiments with ground truth $\tau_{\mathrm{lt}}=10\;s$ for each of three different sets of Δt and evaluated their results (Figure 4a). To resemble the typical experimental dataset size, around 100 FRET time traces were generated for each Δt. The following interval sets were investigated:

- short intervals: 0.05, 0.1, 0.15, 0.25, 0.4s. These lie on the steep left part of the $\tau_{\mathrm{app}}$ vs. Δt curve given by Equation (1).
- medium intervals: 0.25, 0.5, 1.0, 2.0, 3.0s, which cover the bend of the $\tau_{\mathrm{app}}$ vs. Δt curve.
- long intervals: 2, 3, 4, 5, 6s, which lie on the flat right part of the $\tau_{\mathrm{app}}$ vs. Δt curve.

As shown in the right panel of Figure 4a, medium intervals yield the most precise results ($\tau_{\mathrm{lt}}=\left(9.9\pm 1.0\right)\;s$ mean ± std across the simulated experiments), followed by long intervals ($\tau_{\mathrm{lt}}=\left(10.2\pm 1.4\right)\;s$). Short intervals also permit accurate results, however with worse precision ($\tau_{\mathrm{lt}}=\left(10.4\pm 2.9\right)\;s$). Also note that the fit failed (i.e., did not converge, yielded infinite covariance or a large negative value) for short intervals in two and for long intervals in one of the simulated experiments. Analysis without proper consideration of traces outliving the observation window (conventional MLE) leads to systematic bias towards lower values ((8.5±2.0)s, (8.4±0.7)s, and (9.5±1.2)s mean ± std for the short, medium, and long interval sets, respectively).

In order to determine the effect of the size of the experimental dataset on the results (Figure 4b), we generated 500 experiments consisting of small (about 50 smFRET traces per Δt), medium (around 100 traces; typical experiment), and large datasets (approximately 200 traces). Other parameters were the same as with medium intervals above. Increasing the dataset size increases the precision (small: $\tau_{\mathrm{lt}}=\left(9.8\pm 1.5\right)\;s$, medium: $\tau_{\mathrm{lt}}=\left(9.9\pm 1.0\right)\;s$, large: $\tau_{\mathrm{lt}}=\left(10.0\pm 0.7\right)\;s$). Notably, hardly any outliers occur in medium-sized and large datasets. Especially for the larger datasets yielding higher-precision results, the necessity of proper survival analysis becomes very evident. Without, the interaction lifetime is substantially underestimated ((8.3±1.1)s, (8.4±0.7)s, and (8.4±0.5)s for the small, medium, and large datasets, respectively).

### 3.3. Data Analysis Pipeline

As a prerequisite to the determination of interaction lifetimes, smFRET tracking data needs to be extracted from microscopy recordings. To this end, we developed an analysis pipeline, which we implemented in an efficient and straightforward software application [9]. The software features permits input of the excitation sequence, definition of the emission channels, optional image registration using fiducial markers, bleed-through correction, and single-molecule localization and tracking. As a novum the single-molecule inspection is assisted by automatic detection of photobleaching events and various filter settings. Fully automated single-molecule analysis can yield false tracks due to cellular background, accumulation of ligands by the cells, etc. We therefore found manual inspection in addition to parametric filtering indispensable. The final filtered datasets are subjected to the presented survival analysis. For further information and detailed instructions, please refer to the software’s manual.

### 3.4. Experimental Application: TCR–pMHC Interaction Lifetimes

To challenge our analysis pipeline experimentally, we recorded datasets of two different TCR–pMHC pairs (5c.c7–IE^k^/MCC, AND–IE^k^/MCC), for which previous studies [7,23] have shown interaction lifetimes of different orders of magnitude.

As in aforementioned articles, we used functionalized glass-supported lipid bilayers (SLB) in lieu of antigen-presenting cells (Section 2.3), which allowed for precise control of protein composition and the use of total internal reflection fluorescence (TIRF) microscopy. T cells with fluorescently labeled TCR seeded onto the SLB would attach and bind to the pMHC (Figure 1). These interactions were monitored via smFRET.

The long-lived AND–IE^k^/MCC pairs entailed some experimental challenges. We found a maximum usable recording interval of about 5 s. For longer Δt, signals moved further between frames than the tracking algorithm could cope. Additionally, many signals migrated towards clusters so that they were not distinguishable until the end. Therefore, despite the insights from Section 3.2, our measurements were restricted to the steep, left part of the $\tau_{\mathrm{app}}$ vs. Δt curve.

**Figure 5:**
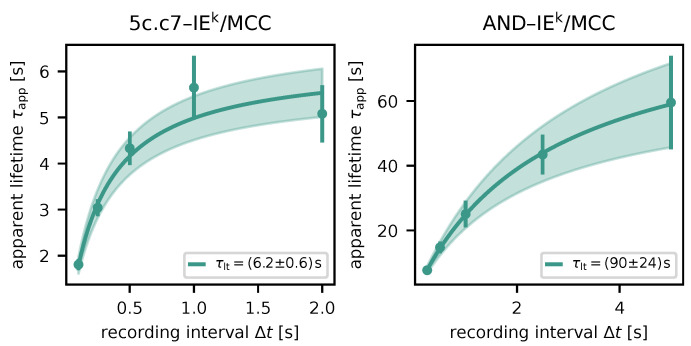
Lifetime measurements for different TCR–pMHC pairs. Apparent lifetimes $\tau_{\mathrm{app}}$ for respective recording intervals Δt are displayed as dots (maximum likelihood estimate via survival analysis) with error bars (standard error of the estimate). The solid line shows the result of fitting Equation (1), the shaded area indicates the uncertainty. (**left**): 5c.c7 TCR, IE^k^/MCC pMHC; (**right**): AND TCR, IE^k^/MCC pMHC.

We determined characteristic lifetimes of (6.2±0.6)s and (90±24)s for 5c.c7–IE^k^/ MCC and AND–IE^k^/MCC, respectively (Figure 5). These are moderately longer than previously published values ((5.0±0.2)s and (80.6±5.9)s) [23], which were derived by measuring the duration of TCR-mediated pMHC immobilization. The difference may be explained by the fact that immobilization data were not analyzed using survival analysis.

## 4. Discussion

Single-molecule FRET assays are a very sensitive tool for the investigation of receptor–ligand interactions as they can capture fast and rare dynamics. In contrast to traditional methods such as surface plasmon resonance and fluorescence cross-correlation spectroscopy measurements, which permit the examination of 3D binding kinetics of dissolved interaction partners, smFRET is used to report on the 2D interaction kinetics of bound receptors and ligands in even the most complex cellular environments [25,26]. Failing to account for side effects of the smFRET image recording process and the limited observation time can lead, however, to a substantial underestimation of the average bond lifetime. We here present an improved and robust statistical analysis tool for the lifetime estimation of receptor–ligand interactions determined by smFRET assays. In order to show the accuracy of the new analysis pipeline, we provide simulated and experimental data and compare the results with the standardized algorithm currently in use by the community.

In particular, we created a new analysis algorithm which robustly estimates apparent (i.e., uncorrected for photobleaching) lifetimes at different frame rates to accurately infer the interaction lifetime. The new feature is based on the mathematical framework of survival analysis. This approach outperforms the original, conventional MLE algorithm, which utilizes solely the number of frames in which the individuals smFRET traces are visible, resulting in better accuracy and robustness.

To test the limits of our method, we used simulated smFRET traces and compared the analysis pipeline’s output to the ground truth. We show that the best results are obtained with intermediate recording delays Δt which cover the curved part of the graph of $\tau_{\mathrm{app}}\left(\Delta t\right)$. If no a priori estimate of the interaction lifetime $\tau_{\mathrm{lt}}$ is available, it is better to err on the long side. However, there are some experimental challenges in this regard: If Δt is greater than $\tau_{\mathrm{lt}}$, few events will be present in more than one consecutive frame since the track lengths are exponentially distributed. Additionally, for large Δt smFRET signals may move by a substantial distance between frames, which can go beyond the single-molecule tracking algorithm’s capabilities. Furthermore, aggregation of molecules can also be a challenge, e.g., during formation of an immunological synapse, in which case it becomes difficult to identify the appearance and disappearance of individual signals. The latter may, to some extent, be mitigated by labeling only a fraction of the receptors or ligands; on the other hand, this reduces the overall dataset size. Comparing the results from simulated datasets of different sizes revealed that in order to obtain reliable results, one should gather at least 50 smFRET traces for each Δt.

Our mathematical model (Section 3.1) relies on interaction lifetimes being exponentially distributed. This implies exponential distribution of the apparent lifetimes, for whose likelihood we can specify an analytic expression due to being memory-less. For systems with non-exponentially distributed lifetimes, the situation would be more complex. First, the distribution of apparent lifetimes and a relation between $\tau_{\mathrm{app}}$, $\tau_{\mathrm{lt}}$, and Δt akin to Equation (1) needed to be derived. Additionally, the survival analysis of individual $\tau_{\mathrm{app}}\left(\Delta t\right)$ may require an iterative approach (Section 5.2 of [24]).

An alternative strategy for the determination of interaction kinetics in a 2D cellular context is to record receptor-mediated ligand immobilization [23]. While this approach is simpler to implement than smFRET, it is limited to slow unbinding rates in relation to the diffusional motion of the ligand. Note that the evaluation of ligand immobilization entails the same challenges as the assessment of smFRET, i.e., a limited observation window and relatively long recording intervals. Thus, it may be advantageous to apply a survival analysis-based method there as well.

We chose to present our new approach using the TCR–pMHC interaction as an example. However, other receptor–ligand pairs can be investigated as well. For instance, integrin α_V_β_3_-binding to fibronectin [27] as well as histone–DNA interactions [28] have been characterized via smFRET. Our experimental and analytical pipeline can easily be transferred to any receptor-ligand pair of interest if respective FRET binding pairs have been established. The binding partners need to be site-specifically decorated with a FRET donor and acceptor molecule within a distance of 10 nm (that means the actual molecular distance needs to be in range of the Förster radius $R_{0}$) in order to yield well-defined single-molecule FRET signals. The fluorescent labels can either be introduced via bio-orthogonal conjugation directly to the protein backbone or via antibody labeling (preferably with smaller antibody fragments such as Fab fragments or single-chain fragments, scF_V_). The distance of the fluorophores can be predicted either via utilizing existing crystal structures of the target proteins or via experimental trial. We recommend the use of newer generation fluorophores such as Alexa Fluor 647 and Alexa Fluor 555, as such FRET pairs allow the tracking of longer FRET trajectories before photobleaching. The imaging system needs to be capable of detecting and tracking single molecules on a planar surface over varying exposure intervals.

To support the experimentalist in dealing with large data amounts, we created a software suite for reproducible filtering of the trajectories as well as decreasing subjective bias in choosing single molecule FRET events. The standard procedure so far was to manually inspect the recorded video sequences for FRET signal appearance and disappearance and noting down the number of frames. The chosen trajectories could not be reinspected and shifting perspective and sensitivity of the analyst in choosing trajectories led to biased data selection. Re-analyzing the data set by a second experimentalist often led to different results. In the presented software, datasets and respective filters can now be reinspected and all chosen and rejected data points can be used for subsequent evaluation. Additionally, basic parameters (such as average intensity of the signal, and background level) associated with each trajectory can be used for automatic filtering. Intensity profiles of the automatically detected single molecules are displayed for a less biased evaluation of single arrival and departure steps of the signal (which are hallmarks of single-molecule signals), disturbance within the track by other single molecules or intensity fluctuations (which could indicate rebinding events), or the premature end of the trajectory because of receptor clustering. Furthermore, the use of automated algorithms (localization, tracking, intensity step detection, …) drastically decreases the amount of time required for analysis. The software is published under a permissive open-source license and thus freely available [9]. We anticipate that our software will facilitate and improve forthcoming interaction lifetime measurements.

In the future, the software may be further improved by deep learning techniques. A neural network could potentially be trained to carry out the final decision whether a smFRET trajectory is accepted for further analysis or not, which is currently a time-consuming task. Datasets previously analyzed by humans could be used as training data. However, since experimental outputs can vary greatly depending on the cells used, imaging conditions, etc., we expect that implementing a deep learning model will be a challenging undertaking.

The bond lifetime of a TCR–pMHC pair is a meaningful parameter for a multitude of scientific questions and, therefore, of interest for a wide scientific community. The threshold time an interaction needs to trigger productive downstream signaling is an important optimization parameter for artificial T cell receptors and equivalents [29,30]. For a small subset of mainly transgenic TCRs these interaction dynamics have been characterized and are used as model systems for other biological questions, yet more and more researchers inquire the binding dynamics of the natural occurring TCR repertoire as well. From lessons from the kinetic proofreading model [31] we know that downstream cascades initiate within a few seconds of pMHC ligation [1,32]. Consequently, the TCR decision process for discrimination of varying ligand potencies happens within this small time window. The direct observation of ligand binding events is, therefore, a very informative tool for understanding T cell immune surveillance (i.e., scanning parameters, triggering thresholds, as well as signaling consequences).

Receptor–ligand bonds in intercellular interactions are typically subjected to an abundance of mechanical cues: tension, shear stress, stretching, compression, etc. [25], all of which can influence the binding kinetics. Bond lifetime measurements therefore may provide valuable information about underlying physical processes.

## Data Availability

The original data presented in the study are openly available in TU Wien Research Data at https://doi.org/10.48436/p2txr-xxy95.

## Supplementary Materials

The following supporting information can be downloaded at: https://www.mdpi.com/article/10.3390/biom14081001/s1, Table S1: Parameters for simulations varying the set of recording intervals; Table S2: Parameters for simulations varying the dataset size; Figure S1: Average numbers of single-molecule FRET traces for simulations varying the set of recording intervals; Figure S2: Average numbers of single-molecule FRET traces for simulations varying dataset sizes; Figure S3: Average numbers of single-molecule FRET traces for experiments.

## Abbreviations

The following abbreviations are used in this manuscript:

APC	antigen-presenting cell
FRET	Förster resonance energy transfer
H57-scF_V_	H57 antibody-derived single-chain fragment
MCC	moth cytochrome C
MHC	major histocompatibility complex
MLE	maximum likelihood estimation
SLB	supported lipid bilayer
SPR	surface plasmon resonance
TCR	T cell receptor
pMHC	peptide-loaded major histocompatibility complex
smFRET	single-molecule Förster Resonance Energy Transfer
