# Supplementary material for: Advanced Quantification of Receptor–Ligand Interaction Lifetimes via Single-Molecule FRET Microscopy
Source: Biomolecules. 2024 Aug 13;14(8):1001. doi: 10.3390/biom14081001 (PMC11352576; doi:10.3390/biom14081001)
Supplement: Supplementary file 1 [file biomolecules-14-01001-s001.zip › biomolecules-3104751-supplementary.pdf]

dataset	τ_{lt} [s]	c_{b}	τ_{off}	start time [s]	Δt [s]	sampling points	simulated traces
short	10	30	1000	2000	0.05	175	11000
					0.10	125	7500
					0.15	100	6000
					0.25	100	4000
					0.40	75	3000
medium	10	30	1000	2000	0.25	125	3100
					0.50	100	2100
					1.00	50	2100
					2.00	30	2100
					3.00	15	3400
long	10	30	1000	2000	2.00	50	1400
					3.00	40	1400
					4.00	30	1600
					5.00	20	2200
					6.00	15	2800

Supplementary Table S1: Parameters for simulations varying the set of recording intervals Δt (fig. 4a)

dataset	τ_{lt} [s]	c_{b}	τ_{off}	start time [s]	Δt [s]	sampling points	simulated traces
small	10	30	1000	2000	0.25	125	1550
					0.50	100	1050
					1.00	50	1050
					2.00	30	1050
					3.00	15	1700
medium	10	30	1000	2000	0.25	125	3100
					0.50	100	2100
					1.00	50	2100
					2.00	30	2100
					3.00	15	3400
large	10	30	1000	2000	0.25	125	6200
					0.50	100	4200
					1.00	50	4200
					2.00	30	4200
					3.00	15	6800

Supplementary Table S2: Parameters for simulations varying the dataset size (fig. 4b)

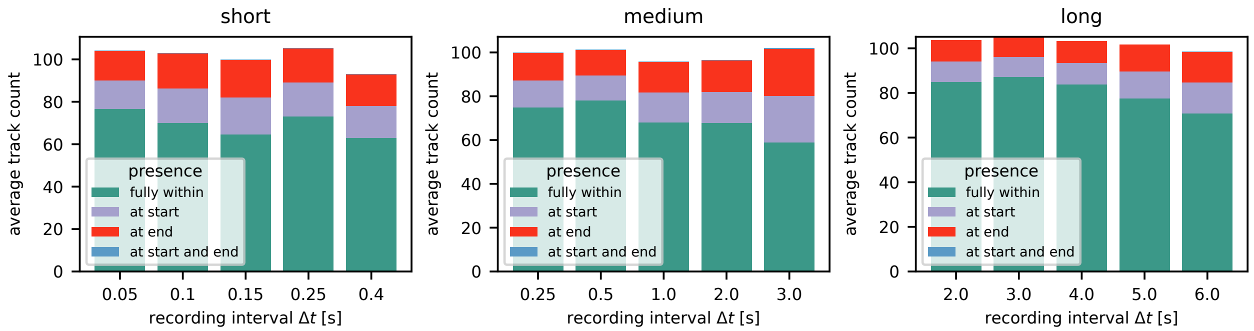

Supplementary Figure S1: Average numbers of single-molecule FRET traces for simulations varying the set of recording intervals Δt (fig. 4 a). Colors indicate whether traces lie fully within the observation window, are already present at the start of the window, still present at the end of the window, or both of the latter two.

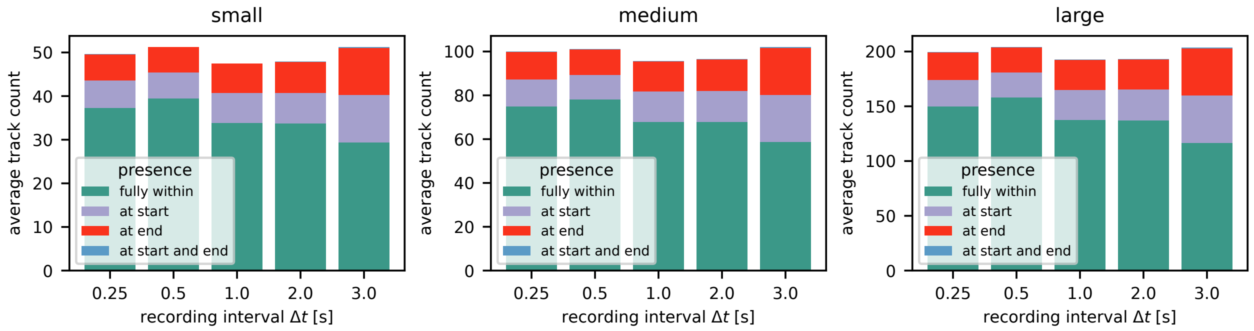

Supplementary Figure S2: Average numbers of single-molecule FRET traces for simulations varying dataset sizes (fig. 4b). Colors indicate whether traces lie fully within the observation window, are already present at the start of the window, still present at the end of the window, or both of the latter two.

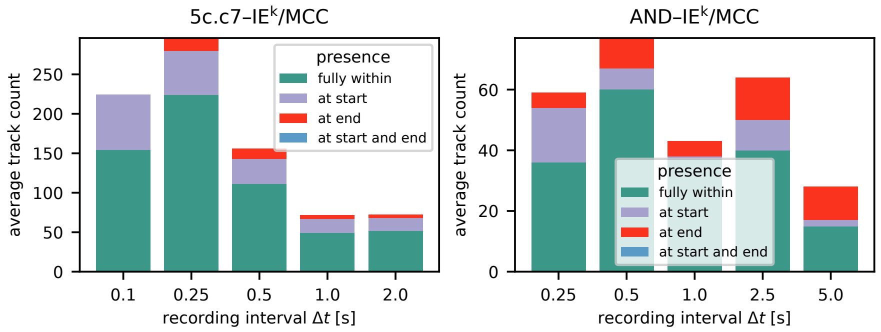

Supplementary Figure S3: Average numbers of single-molecule FRET traces for experiments (fig. 5). Colors indicate whether traces lie fully within the observation window, are already present at the start of the window, still present at the end of the window, or both of the latter two.
